# Archaea influence composition of endoscopically visible ileocolonic biofilms

**DOI:** 10.1080/19490976.2024.2359500

**Published:** 2024-06-02

**Authors:** Elisabeth Orgler, Maximilian Baumgartner, Stefanie Duller, Christina Kumptisch, Bela Hausmann, Doris Moser, Vineeta Khare, Michaela Lang, Thomas Köcher, Adrian Frick, Markus Muttenthaler, Athanasios Makristathis, Christine Moissl-Eichinger, Christoph Gasche

**Affiliations:** a Division of Gastroenterology and Hepatology, Department of Internal Medicine 3, Medical University of Vienna, Vienna, Austria; b Diagnostic and Research Center for Molecular BioMedicine, Diagnostic and Research Institute of Hygiene, Microbiology and Environmental Medicine, Medical University of Graz, Graz, Austria; c Department of Medicine II, University Hospital, Munich, Germany; d Centre for Microbiology and Environmental Systems Science, Joint Microbiome Facility of the Medical University of Vienna and the University of Vienna, Vienna, Austria; e Division of Microbiology, Department of Laboratory Medicine, Medical University of Vienna, Vienna, Austria; f Department of Cranio-Maxillofacial and Oral Surgery, Medical University of Vienna, Vienna, Austria; g Metabolomics Service and Research Facility, Vienna Biocenter Core Facilities, Vienna, Austria; h Faculty of Chemistry, Institute of Biological Chemistry, University of Vienna, Vienna, Austria; i Institute for Molecular Bioscience, The University of Queensland, Brisbane, Australia; j Loha for Life, Center for Gastroenterology and Iron Deficiency, Vienna, Austria

**Keywords:** IBS, UC, bile acids, biofilm, archaea, *Methanobrevibacter*, facultative anaerobes

## Abstract

The gut microbiota has been implicated as a driver of irritable bowel syndrome (IBS) and inflammatory bowel disease (IBD). Recently we described, mucosal biofilms, signifying alterations in microbiota composition and bile acid (BA) metabolism in IBS and ulcerative colitis (UC). Luminal oxygen concentration is a key factor in the gastrointestinal (GI) ecosystem and might be increased in IBS and UC. Here we analyzed the role of archaea as a marker for hypoxia in mucosal biofilms and GI homeostasis. The effects of archaea on microbiome composition and metabolites were analyzed via amplicon sequencing and untargeted metabolomics in 154 stool samples of IBS-, UC-patients and controls. Mucosal biofilms were collected in a subset of patients and examined for their bacterial, fungal and archaeal composition. Absence of archaea, specifically *Methanobrevibacter*, correlated with disrupted GI homeostasis including decreased microbial diversity, overgrowth of facultative anaerobes and conjugated secondary BA. IBS-D/-M was associated with absence of archaea. Presence of *Methanobrevibacter* correlated with *Oscillospiraceae* and epithelial short chain fatty acid metabolism and decreased levels of *Ruminococcus gnavus*. Absence of fecal *Methanobrevibacter* may indicate a less hypoxic GI environment, reduced fatty acid oxidation, overgrowth of facultative anaerobes and disrupted BA deconjugation. Archaea and *Ruminococcus gnavus* could distinguish distinct subtypes of mucosal biofilms. Further research on the connection between archaea, mucosal biofilms and small intestinal bacterial overgrowth should be performed.

## Introduction

Irritable bowel syndrome (IBS) is the most common chronic gastrointestinal (GI) disorders with an estimated prevalence of 9–12% in European and North America countries.^1,2^ Symptoms are persistent and include abdominal pain in combination with diarrhea (IBS-D), constipation (IBS-C) or mixed altered bowel movements (IBS-M).^3^ Disease pathophysiology remains poorly understood and causative treatment options are currently lacking, frustrating patients and physicians alike. Similar to IBS, the prevalence of inflammatory bowel diseases (IBD) has been increasing, with a current estimated prevalence of 0.5% in Western countries and incidences further rising in newly westernized countries.^4^ In both Crohn’s disease (CD) and ulcerative colitis (UC) chronic inflammation damages the intestinal mucosa leading to diarrhea, abdominal pain, bleeding and ulceration.^5,6^ Additionally, chronic inflammation, oxidative DNA damage and insufficient DNA repair significantly increase the risk of colorectal cancer in IBD patients.^7–11^

Even though pathogenesis for both IBS and IBD remains incompletely understood, amounting evidence points at the intestinal microbiome as a one of the major players in disease pathophysiology.^12–14^ Distinct changes in microbial composition and reduced diversity have been demonstrated in IBD and IBS.^15,16^ One exemplary piece of evidence of the significance of microbiota in disease pathophysiology, is the efficient transient symptom alleviation in IBS and remission-inducing in UC through fecal microbiota transplantation.^17,18^

However, most microbiome studies lack two essential aspects: First, focus is laid on bacteria, neglecting other microbial players such as fungi and archaea, which even though in number less prevalent, just as importantly affect the overall microbial composition.^19,20^ Changes in fungal and archaeal compositions have indeed been found in IBD and IBS.^21–24^ For instance, a decreased fungal diversity and depletion of some archaeal strains, like *Methanobrevibacter*, has been shown in IBS.^12,21^ Furthermore, some archaea have been suggested as contributors to prolonged colonic transit time, with a protective potential against diarrhea.^25^ Secondly, research on spatial distributions of microbes in the intestinal tract is scarce with most studies focusing only on the overall fecal composition.^26^ A gradient in oxygen levels, with a high concentration in the upper GI tract, decreasing until a minimal concentration in the colon, is responsible for site-specific microbial profiles in physiologic conditions.^27^ This is observable in the presence of facultative anaerobes, bacteria capable of using oxygen for energy production, which are highly abundant in the small intestines and largely depleted in the colon.^28^ Inversely, obligate anaerobes are the predominant inhabitants of the colon, where no oxygen is available.^29^ This phenomenon is controlled by the host via regulation of oxygen and other electron acceptor’s availability.^27^ If these host control mechanisms are impaired, colonic oxygen levels rise and a bloom in facultative anaerobes follows, causing colonic dysbiosis.^30^ High abundance of facultative anaerobes in the colon is a common feature in IBS, UC and colorectal cancer.^30^ This phenomenon may most likely not only affect bacteria, but might encompass archaea, which are strictly anaerobes,^19^ and fungi as well. Causes of disturbances of the host’s control capacity are incompletely understood, but might include a high-fat, high-sugar diet, antibiotics and infection with enteropathogens – known risk factors for IBS and IBD.^30^

Only recently, a novel feature has been described in IBS and UC with potential pathophysiologic importance: endoscopically visible biofilms, consisting of bacteria and adhering to the mucosal wall, which may affect intestinal homeostasis.^31^ Biofilms are a unique mode of growth, in which microbes attach tightly to each other and a surface and produce extracellular matrix, thereby forming a micro-ecosystem protecting them from outer stressors.^32–34^ Bacterial biofilms are usually induced in hostile environments and have been recognized in many other human diseases, like endocarditis or implant infections,^33^ however, have only now been recognized as potential contributors to GI diseases. We previously showed that biofilms can be found in 57% of IBS, 34% of UC and 22% of CD patients, whereas only 6% of healthy controls harbored biofilms.^31^ Scanning electron microscopy of biofilms showed high bacterial density, adherence and partly invasion of bacteria into the epithelium.^31^ We further detected reduced bacterial diversity in patients harboring biofilms and disease-specific compositions. Metabolomic analysis revealed an approximately ten-fold increase in bile acids (BA) in biofilms and matched stool samples.^31^ Biofilms also correlated with a bloom of *Ruminococcus gnavus*, which was proved to be a potential biofilm former *in vitro*.^31^ IBS-D had been associated with high BA-levels in stool before, which is hypothesized to be partially responsible for the disorder.^35^

Here we studied such biofilms and stool samples of IBS, UC and control patients for the presence of fungi and archaea to determine their role in biofilm composition and examine their effect on the abundance of facultative anaerobes. We further investigated bacterial metabolites with untargeted metabolomics and targeted BA analysis to understand metabolomic differences among disease groups. Thereby, the present study enhances understanding of the interplay between archaea, bacteria and host metabolism in the context of IBD and IBS.

## Results

### Absence of archaea is associated with gastrointestinal disease and reduced bacterial diversity

We previously portrayed endoscopically visible bacterial biofilms in the ileum and colon in patients with IBS and UC.^31^ Here we asked to which extent archaea or fungi participate in such biofilm production. Fecal DNA of 154 individuals (96 IBS, 18 UC and 40 controls), 85 with and 69 without biofilms was subjected to a nested qPCR for the archaeal 16S region.

A threshold of a Ct value of 30 cycles in qPCR was set to distinguish archaea positive (archaea-pos) from archaea negative (archaea-neg) stool samples in two patient cohorts (Supplementary Table S1 and S2). Applying this threshold, archaea were detected in 50/154 stool samples (32.5%). To investigate the association of archaeal presence in stool with GI disease, endoscopically visible mucosal biofilms or intake of microbiome modulating drugs such as proton pump inhibitors (PPI) and antibiotics, a multivariate logistic regression model was applied.

There was no difference in archaea-status between the two patient cohorts (*p* = .84, logistic regression). Archaea were reduced in patients with IBS-D/-M (diarrhea or mixed subtype) (OR 0.28, *p* < .05) and there was a trend in UC (OR 0.27, *p* < .08) and IBS-C (OR 0.23, *p* = .06). Endoscopically visible biofilms were not correlated to archaea status: 58.7% of subjects in the archaea-neg group had endoscopic biofilms compared to 48% of archaea-pos patients (*p* = .23, Fisher’s exact). In terms of medication history, there was a slight trend toward absence of archaea in patients with exposure to proton pump inhibitors (PPIs) (OR 0.52, *p* = .18; Table 1). Antibiotic intake was not associated with archaeal status. Taken together, absence of archaea correlated with GI disease states with altered microbiome composition such as IBS and UC.

**Table 1.** GI disease correlates with decreased fecal archaea.

	Archaea pos		Archaea neg		Odds ratio	2.5/97.5% interval	p-value
*Categorization across disease groups*							
Controls	18/40	(45.0%)	22/40	(55.0%)			
**Irritable bowel disease D/M**	**20/75**	**(26.7%)**	**55/75**	**(73.3%)**	**0.29**	**(0.09–0.88)**	**0.03**
Irritable bowel disease C	7/21	(33.3%)	14/21	(66.7%)	0.24	(0.05–1.02)	0.06
Ulcerative colitis	5/18	(27.8%)	13/18	(72.2%)	0.27	(0.06–1.11)	0.08
*Categorization across patient characteristics*							
Antibiotic intake	24/36	(66.7%)	47/68	(69.1%)	1.53	(0.55–4.60)	0.43
PPI intake	13/34	(38.2%)	33/69	(47.8%)	0.53	(0.20–1.35)	0.18
Biofilm presence	24/50	(48.0%)	61/104	(58.7%)	0.69	(0.25–1.82)	0.44

Multivariate Logistic Regression of Archaea Status relative to Controls (above). Multivariate Logistic Regression of Archaea Status relative to medication and biofilm presence (below): intake of medication depending on archaeal status, group sizes vary due to available medication histories.

**Figure 1.**
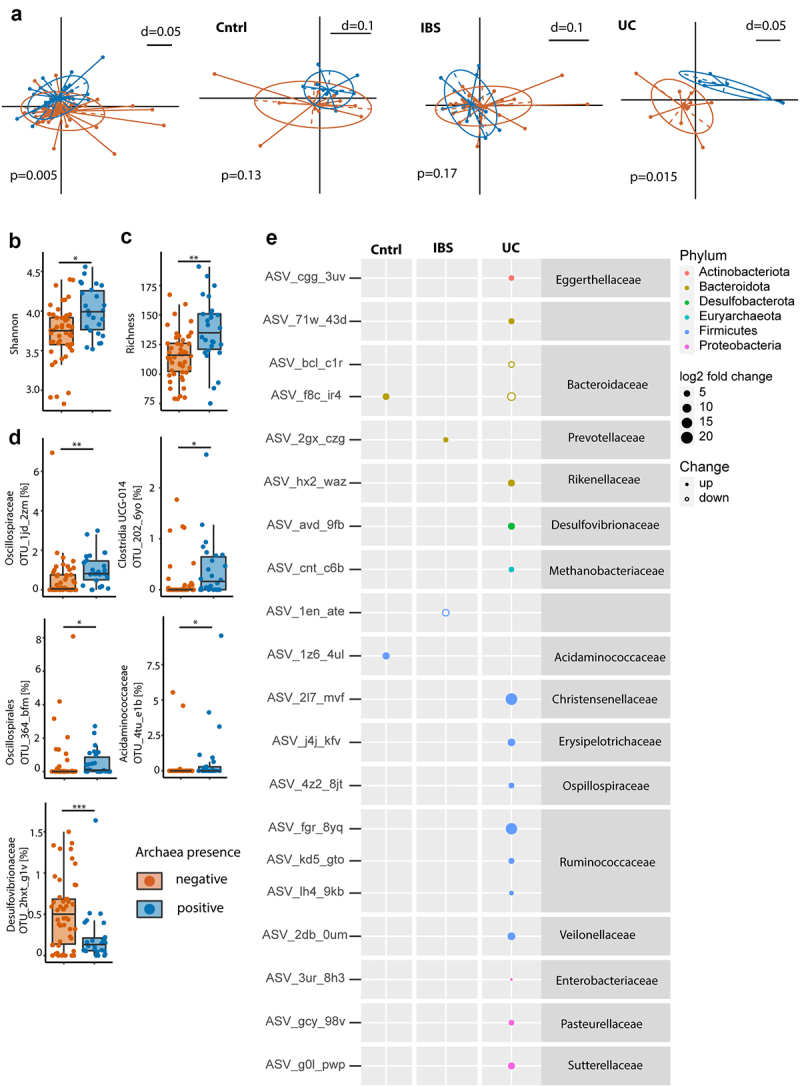
Absence of archaea in stool correlates with decreased microbial diversity. (a–e) Comparison between stool samples with archaea (archaea-pos, blue) and without archaea (archaea-neg, orange) detected via qPCR in patient cohort 1. (a) NMDS plots of generalized unifrac distances of bacterial composition in all samples, controls, IBS- and UC-patients (from left to right), determined via 16S-rRNA sequencing. (b) Bacterial diversity as defined by Shannon’s index. (c) Bacterial richness. (d) Relative abundance of differential abundant bacterial OTUs. (e) Amplicon sequencing variant-based differences between archaea-high and archaea-low stool samples. Size of dots represent fold change, full-dots represent up-regulation in archaea positive samples, empty dots represent down-regulation. Dots are colored based on bacterial phylum. Statistical analysis: cohort 1, total *n* = 76 stool samples (50 archaea-neg, 26 archaea-pos; 23 controls, 37 IBS- and 16 UC-patients). (a) PERMANOVA of distance matrices. (b–d) Kruskal-Wallis rank sum test, with Benjamini-Hochberg correction for multiple comparisons. (e) DESeq2, only significant findings (*p* < .05 after correction for multiple comparisons) are shown. *p ≤ .05, **p ≤ .01.

IBS, UC and control patients stemmed from two cohorts: cohort 1 was used for exploration and cohort 2 was established as a confirmation cohort for microbiome analysis. To gain a better understanding of microbiome changes depending on archaeal status, we performed 16S rRNA gene amplicon sequencing of DNA from stool samples of IBS, UC and control patients in cohort 1. Stool samples were compared depending on their archaeal status (archaea-pos versus archaea-neg as assessed via qPCR). Overall, there was significant clustering of microbiome compositions depending on archaeal status (Figure 1a). Archaea influenced the fecal microbiome composition independently from the presence of biofilms (Suppl. Figure S1). Within the subgroups, UC patients showed significant bacterial signatures clustering depending on archaeal status (PERMANOVA *p* = .02 in UC), whereas in controls and IBS patients, there was no significant difference at the given sample number (*p* = .13 in controls and *p* = .17 in IBS).

Archaea-neg stool samples revealed a significantly reduced bacterial richness and diversity (Figure 1b,c). Furthermore, OTUs belonging to, *Oscillospirales, Acidaminococcaceae, Clostridia UCG 014* and *Oscillospiraceae* were enriched in archaea-pos stool samples. An OTU belonging to *Desulfovibrionaceae* showed decreased relative abundance in archaea-pos stool samples (Figure 1d). Subgroup analysis at the ASV level confirmed differences in bacterial composition dependent on archaea status in UC and minor changes in controls as well as IBS, further illuminating the underlying alterations differentiating the disease groups from each other (Figure 1e). For instance, archaea-pos UC samples showed an up-regulation of ASVs belonging to *Ruminococcus, Oscillospiraceae, Eggerthellaceae, Rikenella-ceae, Desulfovibrionaceae, Methanobacteriaceae, Christensenellaceae, Erysipelotrichaceae, Veillonellaceae, Enterobacteriaceae, Pasteurellaceae, Sutterellaceae* and a reduction in two ASVs belonging to *Bacteroidaceae*. In archaea-pos controls an up-regulation of ASVs belonging to *Bacteroidaceae* and *Acidaminococcaceae* was seen. In stool samples of archaea-pos IBS patients an ASVs belonging to *Prevotellaceae* was increased and a Firmicutes ASV was reduced.

To analyze the correlation between fecal archaea-status and bacterial OTUs independent from disease we generated a linear model using MaAsLin2.^36^ Confirming our previous analysis of cohort 1, there was a significant correlation between OTUs 1jd_2zm (*Oscillospiraceae UCG-002*), 2o2_6yo (*Clostridia*) and 4tu_e1b (*Acidaminococcaceae*) and Archaea-pos (Suppl. Figure S2A). Additionally, there was a correlation between archaea-pos and OTUs belonging to *Eubacterium siraeum group, Marinifila-ceae, Bifidobacterium, Christensenellaceae R-7, Prevotella* and *Peptococcaceae*. Archaea-pos was inversely correlated with OTUs belonging to *Bacteroides, Erysipelotrichaceae* and *Holdemania*. Of all variables used in the model, archaea-status was correlated with most bacterial OTUs (n = 16), followed by UC (n = 10) and IBS-C (n = 6). UC showed a positive correlation with OTUs bel-onging to *Bacteroides*, *Eggerthellaceae* and *Bifidobacterium*. OTUs belonging to *Oscill-ospiraceae, Akkermansia, Ruminococcaceae, Desulfovibrionaceae* and *Lachnoclostridium* were negatively correlated with UC. IBS-C was posit-ively correlated with OTUs belonging to *Christ-ensenellaceae R-7, Victicallis, Oscillospiraceae, Ruminococcaceae* and *Bacteroides*. IBS-M/D was positively correlated with OTUs belonging to *Prevotella* and *Parabacteroides*. In line with our previous findings,^31^ presence of endoscopically visible biofilms had a minor effect on fecal microbiota (Suppl. Figure S2A).

To assess the reproducibility of our findings, we repeated fecal 16S rRNA gene amplicon sequencing and archaea qPCR in the second patient cohort (n = 78, Supplementary Table 2). MaAsLin2 showed a similar pattern with archaea-neg being inversely correlated with OTUs belonging to *Christensenellaceae R-7, Oscillospiraceae UCG-002* and *Eubacterium siraeum* group. Archaea-status also correlated with the highest number of OTUs amongst model-variables. IBS subtypes (-D/-M vs. C) and presence of endoscopically visible biofilms correlated with different OTUs in the second cohort compared to the first (Suppl. Figure S2B). Furthermore, we trained a machine learning classifier on microbiome data of cohort 1 using the qiime2-sample-classifier plugin. Area under the receiver operating characteristic curve was 0.96 for detecting archaea-pos vs. -neg samples. The trained classifier could detect archaea-status with 88% accuracy (Suppl. Figure S2C). When applying the classifier on cohort 2, archaea-status was detected with 74% accuracy (Suppl. Figure S2D).

These results demonstrate that presence of archaea correlates with major microbiota shifts, independent of GI disease. Archaea-neg stool samples are associated with a reduction in bacterial diversity and showed specific alterations in bacterial composition, specifically a decrease in *Oscillospiraceae UCG-002, Christensenellaceae R-7* and *Eubacterium siraeum* group.

### Archaeal absence in stool correlates with a shift in metabolites indicative of reduced SCFA oxidation

**Figure 2.**
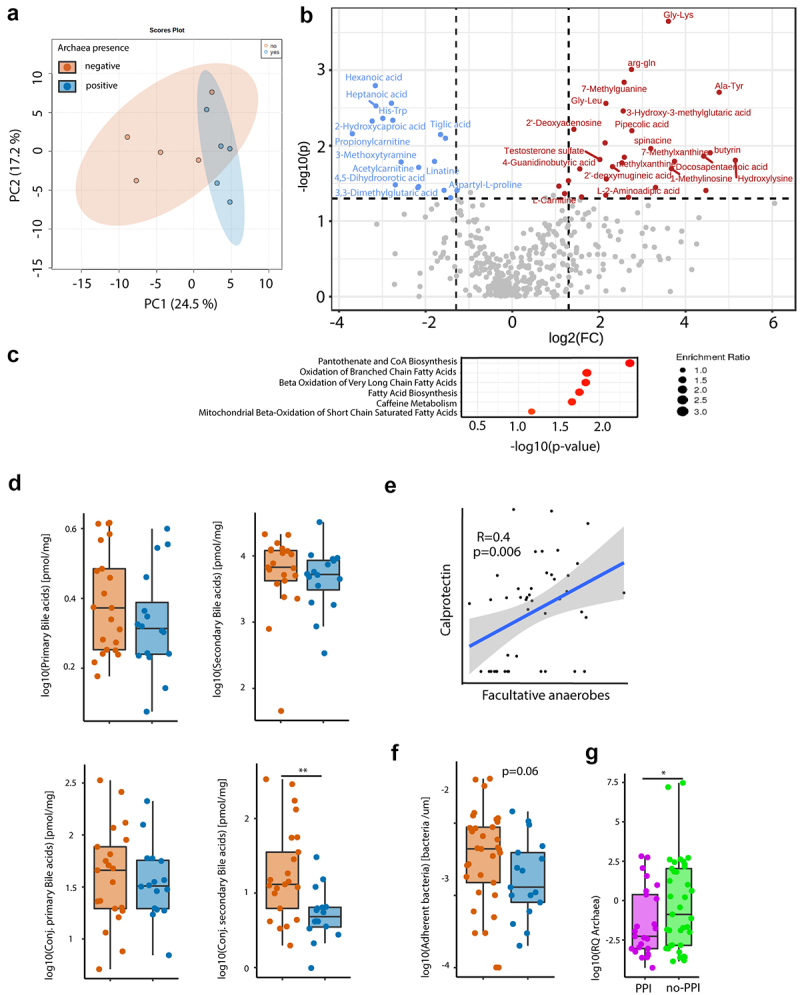
Presence of archaea in stool correlate with SCFA and BA homeostasis. (a,b,d,f) Comparison between archaea-pos (blue) and archaea-neg (orange) stool samples. (a) PCA plot of stool sample metabolite composition. (b) Volcano plot of metabolomics data, p-value threshold 0.05; log2 fold-change threshold ±1. (c) Small Molecule Pathway Database pathway enrichment ratios of metabolomics data. (d) Stool sample bile acid concentrations detected via HPLC-MS. (e) Correlation of fecal calprotectin and relative abundance of facultative anaerobes. (f) Number of bacteria within 3-μm distance from the epithelium detected via DAPI, normalized to length of epithelium per section as determined via confocal microscopy of colonic biopsies. (g) Archaea qPCR RQ values in patients with PPI intake (PPI, purple, *n* = 25) and no PPI intake in the previous five years (no-PPI, green, *n* = 37). Statistical analysis: (a–c) *n* = 5 archaea-pos and 5 archaea-neg stool samples. (d) Mann Whitney U test, *n* = 16 archaea-pos and 21 archaea-neg stool samples. (e) Linear regression analysis, *n* = 47 stool samples, *p ≤ .05; **p ≤ .01.

Archaea are strictly anaerobic and might therefore be used as a surrogate marker for GI hypoxia which is often disrupted in GI diseases.^27,30^ To investigate a connection between the abundance of archaea and shifts in microbiome metabolic function, an untargeted metabolomics approach was applied in a subset of cohort 1. Metabolome analysis of archaea-pos versus archaea-neg stool samples revealed specific metabolomic profiles between the two groups (Figure 2a). A volcano plot of stool metabolites showed an increase of several fatty acids and acetyl-carnitine, which is implicated in mitochondrial energy production, in archaea-pos stool samples (Figure 2b).

Metabolic pathways prediction revealed a significant enrichment of pantothenate and Coenzyme A biosynthesis in archaea-pos samples, a main enzyme involved in synthesis of fatty acids and phospholipids as well as the degradation of the former. A significant increase in fatty acid oxidation was observed in archaea-pos samples. Since we have detected an alteration of bile acid (BA) metabolism in biofilms and stool samples of biofilm-positive IBS patients before, fecal BA composition was analyzed via HPLC-MS. There was a significant increase in conjugated secondary BA in archaea-neg stool samples (Figure 2d).

Calprotectin was measured in stool samples of IBS, UC and control subjects of cohort 1 to evaluate intestinal inflammation. We found a correlation between calprotectin levels and the relative abundance of facultative anaerobes (Figure 2e), indicative of increased luminal oxygen levels upon inflammation that disrupted microbial homeostasis. There was a trend toward higher calprotectin levels in archaea-pos versus archaea-neg subjects (Mann Whitney-U *p* = 0.07). Confocal microscopy and a machine learning-guided imaging analysis pipeline has been used to investigate the concentration of bacteria at the epithelium as previously described.^31^ There was a trend toward increased bacterial mucus invasion, signified by the number of epithelium-adherent bacteria, in patients with archaea-neg stool samples (Figure 2f).

To further investigate the connection of archaea and PPI, the relative quantification (RQ) values of the archaea qPCR were plotted. Confirming the results from the logistic regression, patients that took PPIs in the previous five years had more than ten-fold lower median archaeal RQ values than patients without PPI (Figure 2g).

In summary, absence of archaea correlated with distinct changes in GI metabolism. Fecal metabolic profiles were indicative of reduced mitochondrial SCFA oxidation and decreased microbial BA deconjugation.

### Mucosal biofilm composition differs depending on presence of archaea

**Figure 3.**
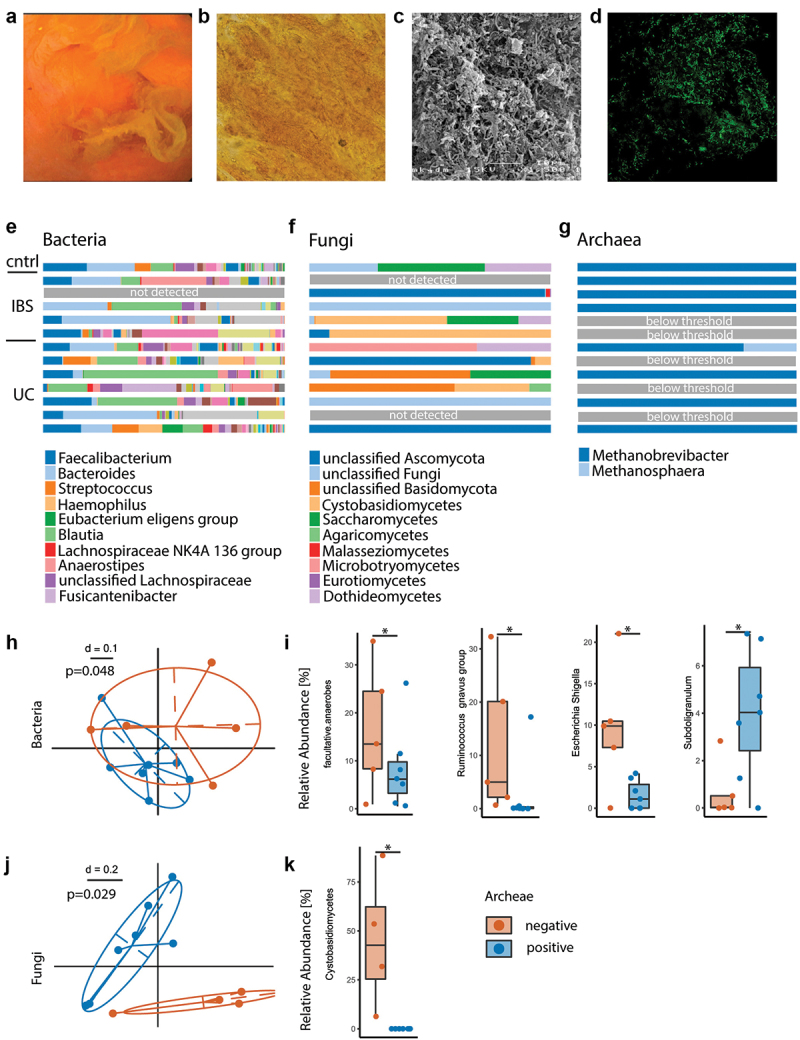
Exploratory analysis of polymicrobial mucosal biofilm composition differs depending on presence of archaea. (a) Example of endoscopic view of mucosal biofilm. (b) Biofilm flush specimen under light microscopy shows yellow color, bacteria and shed epithelial cells. (c) SEM analysis of biofilm flush shows a thick layer of bacterial biofilm and extracellular matrix. (d) FISH with general bacterial probe (green) of methacarn fixed biofilm flush sample. (e–g) Stacked bar plot of relative abundance data, bacteria (e), fungi (f) and archaea (g). (h–k) Comparison between archaea-pos (blue) and archaea-neg (orange) biofilm flush samples. (h) NMDS plot of generalized unifrac distances of bacterial composition. (i) Relative abundance of facultative anaerobes and bacterial genera. (j) NMDS plot of generalized unifrac distances of fungal composition. (k) Relative abundance of Cystobasidiomycetes. Statistical analysis: (e–k) *n* = 13 (1 cntrl, 5 IBS, 7 UC), (h, i) *n* = 7 archaea-pos, 5 archaea-neg. (k) *n* = 6 archaea-pos, 4 archaea-neg. (h) PERMANOVA of the distance matrices, (i,k) Kruskal-Wallis rank sum test. (j) Mann Whitney U test, *n* = 9 archaea-high, 16 archaea-low, *p ≤ .05.

The bacterial composition in mucosal biofilms differs from stool.^31^ To study archaeal and fungal presence within biofilms, endoscopically removed biofilm flushes were collected during colonoscopy and used for high yield microbial DNA extraction. Light microscopy, scanning electron microscopy and fluorescent in-situ hybridization confirmed that these endoscopically collected adherent membranes represent biofilms (Figure 3a–d). Colonic biofilm flushes were collected from a subset of IBS, UC and control patients (*n* = 13) in cohort 1 and underwent gene amplicon sequencing analysis for 16s rRNA gene (bacteria), ITS (fungi) and a two-step nested-PCR targeting the archaeal 16S rRNA gene (archaea). In fact, biofilms harbor not only bacteria, but are polymicrobial communities. To distinguish archaea-pos and archaea-neg biofilm samples, <50% archaea sequencing reads (after nested-PCR targeting the archaeal 16S rRNA gene) relative to bacterial reads stemming from unspecific amplification was defined as archaea-neg. Whilst bacteria and fungi were present in all but one and two samples, respectively; eight out of 13 samples (62%) were archaea-pos. The majority of archaea were classified as *Methanobrevibacter* with only minor amount of *Methanosphaera* and no other detected taxa. Relative abundance of genera between groups are depicted in Figure 3e–g. When comparing archaea-pos versus archaea-neg biofilm samples, we found specific alterations in bacterial composition between the two groups confirming our findings from stool (Figure 3h–k). Non-Metric Multi-Dimensional Scaling (NMDS) plots depicted significant clustering of bacterial biofilm composition depending on archaeal presence (Figure 3h). Bacterial diversity was reduced in archaea-neg biofilms, however these findings did not reach significance (Mann-Whitney-U, *p* = .2). Archaea-neg biofilms had an increase in facultative anaerobic bacteria (Figure 3i), bacteria capable of surviving in both oxygen-enriched and -depleted conditions. Specifically, bacteria belonging to the *Escherichia/Shigella* genus were significantly enriched in archaea-neg biofilms. Additionally, bacteria of the *Ruminococcus gnavus* group showed a bloom in archaea-neg biofilms and were completely depleted in archaea-pos biofilms. Furthermore, archaea-pos biofilms were enriched in short-chain fatty acid (SCFA)-producing bacteria of the *Subdoligranulum* genus. From eight biofilm flushes, we had the matching archaea PCR data of stool samples from the same individuals. With our methodology the match in archaea-status between stool and biofilm was 75%. One patient that had archaea in the biofilm had no detectable archaea in stool, and vice versa for one other patient (Supplementary Figure S3).

Biofilm samples were further evaluated for their fungal composition. Here, we also found alterations between archaea-pos and archaea-neg biofilms, as shown in the NMDS plot in Figure 3j. Specifically, fungi of the *Cystobasidiomycetes* class were enriched in archaea-neg biofilms (Figure 3k).

Taken together, this data demonstrates that polymicrobial mucosal biofilm composition differs depending on archaeal presence and that archaea-neg biofilms show features of overgrowing facultative anaerobes and R. gnavus.

## Discussion

Biofilms are complex, polymicrobial ecosystems, which occur in various natural settings, ranging from hot water springs, to desserts, oceans, and human body niches.^32,33^ In particular, biofilms have recently been recognized in the GI tract, with microscopic biofilms first described in colorectal cancer^37–39^ and only now as endoscopically visible biofilms adhering to the mucosa in IBS and IBD.^31^ This unique mode of growth might reflect a response mechanism to microbial stress or impaired host defense mechanisms. In biofilm positive patients increased bacterial invasion into the mucus layer has been observed.^31^ This concept of biofilms as a means for more intimate contact between microbes and host could likely be a central pathophysiologic mechanism in any disease with barrier dysfunction.^31,40^ Here, we provide further insight into the polymicrobial composition of biofilms including fungi and archaea and demonstrate that *Methanobrevibacter* serves as marker for colonic homeostasis with a predominance of obligate anaerobes.

For this study, DNA extraction methods have been adapted for fungi and archaea and biofilm material has been collected from the colonic lumen after flushing. Archaea-neg biofilms revealed a drastic enrichment in facultative anaerobes and bacteria associated with a disrupted microbiome, such as *Escherichia/Shigella* genus. This trend was confirmed in stool samples, with archaea-neg samples featuring altered bacterial composition and reduced diversity. Archaea-pos stool samples correlated with the anaerobe *Oscillospiraceae*, which include many gut commensals. These findings were further replicated and confirmed in a separate cohort of 78 patients. Furthermore, fungal composition varied widely depending on archaeal status in biofilms. Specifically, *Cystobasidiomycetes* were significantly enriched in archaea-neg biofilms, a fungal class which has been associated with obesity and abnormalities in metabolic markers, such as cholesterol.^41^

As previously described, oxygen levels are usually strictly controlled by the host, with intravascular-like levels in the duodenum, decreasing longitudinally along the GI tract and reaching hypoxic levels in the colon.^27,30^ This mechanism creates different ecological niches along the GI tract, regulating which microbes inhabit the different compartments.^29^ At the center of the control mechanism are mitochondria, which deplete intraluminal oxygen in the colon via oxidative phosphorylation coupled to fatty acid oxidation.^30^ Physiologically, facultative anaerobes occur in the small intestine, where they are at an advantage with their capacity to use oxygen and nitrate for energy production, and are scarce in the colon.^29,30^ However, if the host loses its regulatory functions (such as during intestinal inflammation), oxygen levels may rise and allow facultative anaerobes to overpopulate the colon.^27^

The anaerobe *Methanobrevibacter* can only populate a hypoxic colon, explaining why its absence correlates with an increase of facultative anaerobes. Metabolomic analysis revealed an increase in fatty acid oxidation in archaea-pos stool samples, an indicator for functioning host control over oxygen availability. *Methanobrevibacter* might therefore serve as marker for colonic homeostasis and its absence might indicate increased luminal oxygen levels. Biofilms were found in archaea-pos and -neg patients and controls, raising the question if biofilms are always pathologic or can also reside in an otherwise balanced microbiome. This is further fueled by the result of our previous study, in which biofilms were present in 57% of IBS, 34% of UC patients but also 6% of healthy controls.^31^ To determine the effects of biofilm-subtypes on the mucosa and if archaea could help distinguish pathophysiologic functions of biofilms, further studies are needed.

Interestingly, the risk factors for loss of oxygen control mechanisms are strikingly similar to those of IBS and IBD, including microbiome altering medications, infections with enteropathogens, high-fat diet and processed-foods.^3,5,6,30^ A central pathomechanism revolves around saturated fatty acids in high-fat foods, which induce oxidative stress in mitochondria and thereby impair their ability to limit colonic oxygen levels.^42,43^ A central bacterium in the context of biofilm formation might be *R. gnavus*, a known mucus degrader^44^ and as we have shown before, an *in vitro* biofilm former, specifically prominent in UC biofilms.^31^ In accordance to these findings, *R. gnavus* was enriched in archaea-neg biofilms in this study. We hypothesize that *R. gnavus* might facilitate erosion of the mucus layer and precedes or allows mucosal biofilm formation. Likely, other factors such as the host’s immune response influence biofilm formation and composition. Of special interest in this context are BA. We found an increased concentration of conjugated secondary BA in archaea-neg stool samples, indicating disrupted bacterial deconjugation. Archaea and some bacteria are capable of de-conjugating bile salts by bile salt hydrolases, which might explain the increase of conjugated BA in archaea-neg samples.^45^ BA have been shown to induce co-aggregation and adhesion mechanisms in bacteria,^46^ thereby potentially further triggering biofilm formation. The connection between absence of archaea, colonic oxygen levels, *R. gnavus*, mucosal biofilms, and bile acid malabsorption warrants further investigation. Finally, our data show different bacterial composition of stool samples depending on archaeal presence in UC.

Based on the aforementioned data we conclude that mucosal biofilms are diverse, with *Methanobrevibacter* being a potential marker for a hypoxic GI environment. Increased abundances of methane and *Methanobrevibacter smithii* have also been connected to IBS-C or small intestinal bacterial overgrowth (SIBO).^22,47^ Besides hydrogen, small intestinal methane production is frequently observed in SIBO patients, which largely overlap with IBS cohorts.^47,48^ Here we found that archaea were significantly reduced in IBS-D/-M patients compared to controls, whilst IBS-C surprisingly also showed a trend toward reduction of archaea. IBS may encompass heterogeneous disease entities. Surprisingly, in this study, IBS fecal bacterial composition was not influenced by archaea.

A drawback of this study is the exploratory scope with a limited sample size and complex disease populations. Particularly in the UC group, a larger sample size to compare clinical features (disease activity, location, therapy) could have increased the outcome. In addition, more detailed medication history including more specific timelines on antibiotic, probiotic and PPI intake could have been useful in interpretation. Further large-scale epidemiological studies should be performed to entangle the interaction of BA metabolism, SCFA oxidation, archaea, SIBO and biofilm formation. Additionally, what remains to be seen, is the cellular effect of different biofilm types and their link to the inflammatory reaction in the mucosa. In conclusion, our data offer insight into polymicrobial biofilm composition and demonstrate that absence of archaea and overgrowth of *R. gnavus* could serve as markers for a disrupted colonic homeostasis with overgrowing facultative anaerobes, reduced mitochondrial SCFA oxidation and a disrupted BA metabolism. Further studies are needed to examine the interaction between fungi, archaea and mucosal biofilms, and understand their downstream effects on the underlying mucosa.

## Patients and methods

### Patient cohort

Biofilms were defined as endoscopically visible adherent layers on the intestinal wall, despite adequate polyethylene glycol (PEG) – based bowel preparation, which either firmly stick to the mucosa or detach in a film-like manner when jet washing is performed.^31^ Patients included had to have high-volume PEG preparation and the colonoscopy appointment had to occur the next day between 8 AM and 1 PM. Bowel preparation was scored with the Boston Bowel Preparation Scale and patients with a score below 6 or with non-PEG-based preparations were excluded. Biofilms were endoscopically removed from the cecum or ascending colon (colonic biofilms) using a jet washer using sodium chloride 0.9% and collected in a 10 ml sterile suction tube. Biofilms termed as “ileal” were located in the terminal ileum and were not sampled for this study. Stool samples were collected the day before endoscopy. Both biofilms and stool samples either processed immediately or frozen at − 80°C.

Some stool samples were available from our previous study (cohort 1, *n* = 76 patients, 16 UC, 37 IBS, and 23 controls; 37 biofilm positive, 39 biofilm negative),^31^ and a new cohort was established (cohort 2, *n* = 78 patients, 2 UC, 59 IBS, and 17 controls). A detailed description of patients characteristics can be found in Supplementary Table S1 and S2. IBS patients were further characterized into the subgroups IBS-C versus IBS-D/-M for patients with either diarrhea or mixed symptoms. IBS-D/-M were combined because of often difficult clinical distinguishability and hypothesized pathophysiologic similarity. Patients were asked about antibiotic, probiotic and PPI intake in the last five years prior to inclusion in a questionnaire. Patients who had taken antibiotics three months prior to colonoscopy were excluded. For the detection of fungi and archaea (which are more difficult to lyse than bacteria) from mucosal biofilms we found that a modified IHMS DNA extraction protocol Q, with intense bead beating (6500 rpm for one minute at a time, repeated 16 times) worked best. DNA was similarly extracted from biofilm flushes (*n* = 13, with 7 UC, 5 IBS, and 1 control) because of higher yield in bacterial, fungi and archaea DNA and reduced contamination with human DNA, which may impair the nested PCR approach for archaea detection.

### Microbiota composition analysis

To screen for fungal presence in samples, PCR for the Internal Transcribed Spacer (ITS) region was performed, using the primers ITS1-30F (primer sequence 5′ to 3′ GTCCCTGCCCTTTGTACACA) and ITS1- 217 R (primer sequence 5′ to 3′ TTTCGCTGCGTTCTTCATCG) with 2X GoTaq Green Master Mix (Promega, USA) and 40 cycles. To detect archaeal DNA, nested PCR for the archaeal 16S rRNA gene was performed as described previously.^25^ Due to elevated cycle number in the nested PCR design, a threshold of a Ct value of 30 cycles in qPCR was set to distinguish archaea positive (archaea-pos) from archaea negative (archaea-neg) samples. Methodology is described in more detail in the supplementary method section.

PCR products were cleaned-up using AMPure XP beads (VWR International, Radnor, USA). Sequencing was performed according to the standard 16S amplicon Sequencing Library Preparation Protocol and MiSeq technology (Illumina, San Diego, USA). Fungal and bacterial sequencing were performed at the laboratory of Clinical Microbiology (Department of Clinical Microbiology, Medical University Vienna). Sequencing of archaeal 16S rRNA genes was performed at the Core Facility Molecular Biology at ZMF, Medical University of Graz.

Analysis of amplicon sequence variants (ASVs) was conducted with DADA2, followed by SINA for taxonomic classification in R. For verification of fungal taxonomy, the most abundant reads were double-checked via BLAST of NCBI. Differences in microbial composition and correlations were analyzed with modified Rhea scripts. Non-metric Multi-Dimensional Scaling was used for visualization of generalized Unifrac distances. To assess cluster significance, permutational multivariate analysis of variance was used. To compare relative abundance of taxa, Kruskal-Wallis Rank Sum Test was applied and to adjust p-values for multiple comparisons, Bonferroni correction was used. Differential abundant ASVs were detected using DESeq2 as described previously.^20^ Linear models were build using MaAsLin2^36^ with the following syntax: microbiome composition ~ presence of arachae + disease + presence of endoscopic biofilms. Machine learning classification was performed using qiime 2 and the sample-classifier plugin. Classify-samples pipeline was run on the genus table of cohort 1 with random forest as model, 100 estimators, 35 folds and 20% of samples as test data. The resulting model was applied to the genus table of cohort 2. All custom code and input tables for the microbiome analysis have been deposited at github: github.com/MaximilianBaumgartner/biofilm_archaea.

### Metabolomic analysis

Metabolomic profiles were analyzed further with the MetaboloAnalyst 5.0 pipeline using standard parameters and enrichment analysis with the SMPDB database.

### Calprotectin analysis

Fecal calprotectin was measured using a validated commercial enzyme-linked immunosorbent assay (BÜHLMANN fCAL ELISA, BUHLMANN Diagnostics).

## Supplementary Material

Supplemental Material

## Data Availability

16S rRNA gene amplicon sequencing data was deposited under the BioProject accession number PRJNA644520 (cohort 1) and PRJNA1100194 (cohort 2). Metadata for all samples, custom code and input tables can be accessed at github: github.com/MaximilianBaumgartner/biofilm_archaea.
