# Supplementary material for: Archaea influence composition of endoscopically visible ileocolonic biofilms
Source: Gut Microbes. 2024 Jun 2;16(1):2359500. doi: 10.1080/19490976.2024.2359500 (PMC11152093; doi:10.1080/19490976.2024.2359500)
Supplement: Supplemental Material [file KGMI_A_2359500_SM1202.zip › Supplements_revised clean.docx]

**Appendices / Supplements**

**Supplementary Table 1: Patient characteristics of cohort 1.**

|  |  | Controls | IBS | p-value  vs. Controls | UC | p-value  vs. Controls |
| --- | --- | --- | --- | --- | --- | --- |
| Total patients |  | 23 | 37 |  | 16 |  |
| Mean age (yr) |  | 52.3  (45-59) | 45.1  (35-55) | 0.13 | 45.3  (35-57) | 0.13 |
| Sex | Female (%)  Male (%) | 13 (56.5 %)  10 (43.5 %) | 26 (70.3 %)  11 (29.7 %) | 0.4 | 7 (43.8 %)  9 (56.3 %) | 0.5 |
| Disease subclassification | IBS-D (%)  IBS-C (%)  IBS-M (%)  No data (%) | n/a | 11 (29.7 %)  3 (8.1 %)  21 (56.8 %)  2 (5.4 %) |  | n/a |  |
|  | Montreal E1 (%)  Montreal E2 (%)  Montreal E3 (%) | n/a | n/a |  | 1 (6.3 %)  6 (37.5 %)  9 (56.3 %) |  |
| Archaea | Archaea pos (%)  Archaea neg (%) | 10 (43.5 %)  13 (56.5 %) | 11 (29.7 %)  26 (70.3 %) | 0.4 | 5 (31.3 %)  11 (68.8 %) | 0.5 |
| Antibiotic intake* | No (%)  Yes (%)  No data (%) | 12 (52.2 %)  6 (26.1 %)  5 (21.7 %) | 11 (32.4 %)  25 (67.6 %)  1 (2.7 %) | 0.02 | 6 (37.5 %)  8 (50.0 %)  2 (12.5 %) | 0.28 |
| PPI intake * | No (%)  Yes (%)  No data (%) | 13 (56.5 %)  6 (26.1 %)  4 (17.4 %) | 15 (40.5 %)  17 (45.9 %)  5 (13.5 %) | 0.16 | 12 (75.0 %)  4 (25.0 %)  0 (0 %) | 0.72 |
| Probiotics intake* | No (%)  Yes (%)  No data (%) | 21 (91.3 %)  0 (0 %)  2 (8.7 %) | 26 (70.3 %)  8 (21.6 %)  3 (8.1 %) | 0.02 | 10 (62.5 %)  6 (37.5 %)  0 (0 %) | 0.003 |
| Calprotectin level | Median (µg/g stool) | 0.51  (36-246) | 21.7  (0-44) | 0.11 | 81.0  (0-26) | 0.001 |
| Biofilm presence | Overall (%)  Subtype ileal (%)  Subtype colonic (%) | 7 (30.4 %)  6 (26.1 %)  6 (26.1 %) | 21 (56.8 %)  16 (43.2 %)  17 (45.9 %) | 0.06 | 9 (56.3 %)  5 (31.3 %)  7 (43.8 %) | 0.18 |

*within the last five years

Values are presented as median (first and third quartile) for continuous variables and n (%) for categorical variables. Mann-Whitney U test and 2-sided Fisher exact test vs. Controls were used to determine P values for continuous and categorical variables, respectively.

**Supplementary Table 2: Patient characteristics of cohort 2.**

|  |  | Controls | IBS | p-value  vs. Controls | UC |
| --- | --- | --- | --- | --- | --- |
| Total patients |  | 17 | 59 |  | 2 |
| Mean age (yr) |  | 57.5  (52-68) | 45.1  (30-56) | 0.007 | 52.5 |
| Sex | Female (%)  Male (%) | 11 (35.5 %)  6 (64.7 %) | 29 (49.2%)  30 (50.8%) | 0.28 | 1 (50 %)  1 (50%) |
| Disease subclassification | IBS-D/-M (%)  IBS-C (%) | n/a | 41 (69.5 %)  18 (30.5 %) |  | n/a |
|  | Montreal | n/a | n/a |  | No data |
| Archaea | Archaea pos (%)  Archaea neg (%) | 8 (47.1 %)  9 (52.9 %) | 16 (27.1 %)  43 (72.9 %) | 0.14 | 0 (0 %)  2 (100 %) |
| Antibiotic intake* | No (%)  Yes (%)  No data (%) | 1 (5.9 %)  5 (29.4 %)  11 (64.7 %) | 3 (5.1 %)  27 (45.8 %)  29 (49.2 %) | 0.53 | 0 (0 %)  0 (0 %)  2 (100 %) |
| PPI intake * | No (%)  Yes (%)  No data (%) | 3 (17.6 %)  3 (17.6 %)  11 (64.7 %) | 14 (23.7 %)  16 (27.1 %)  29 (49.2 %) | 1 | 0 (0 %)  0 (0 %)  2 (100 %) |
| Probiotics intake* | No (%)  Yes (%)  No data (%) | 4 (23.5 %)  2 (11.8 %)  11 (64.7 %) | 16 (27.1 %)  14 (23.7 %)  29 (49.2 %) | 0.67 | 0 (0 %)  0 (0 %)  2 (100 %) |
| Biofilm presence | Overall (%)  Subtype Ileal (%)  Subtype colonic (%)  No subtype data (%) | 6 (35.3 %)  3 (17.6 %)  0 (0 %)  2 (11.8 %) | 40 (67.8 %)  19 (32.2 %)  6 (10.2%)  15 (25.4%) | 0.02 | 2 (100%)  0 (0%)  0 (0%)  2 (100%) |

*within the last five years

Values are presented as median (first and third quartile) for continuous variables and n (%) for categorical variables. Mann-Whitney U test and 2-sided Fisher exact test vs. Controls were used to determine P values for continuous and categorical variables, respectively.


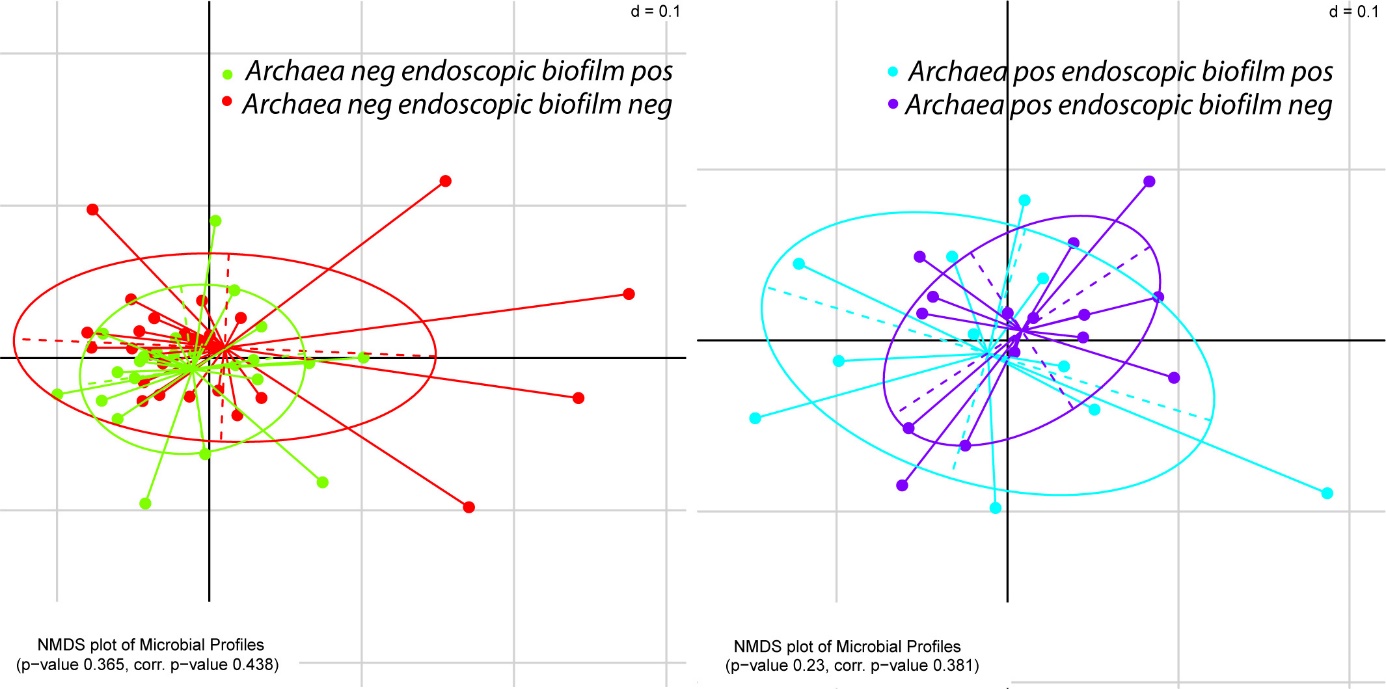


**Supplementary Figure 1: Influence of archaeal absence and presence on fecal microbiome is independent from biofilm status.** NMDS plots of generalized unifrac distances of fecal bacterial composition as determined with 16S-rRNA sequencing in archaea-neg patients with (red, n=24) and without (green, n=26) endoscopically visible biofilms on the left, archaea-pos patients with (turquois, n=11) and without (purple, n=15) endoscopically visible biofilms on the right. PERMANOVA of distance matrices.


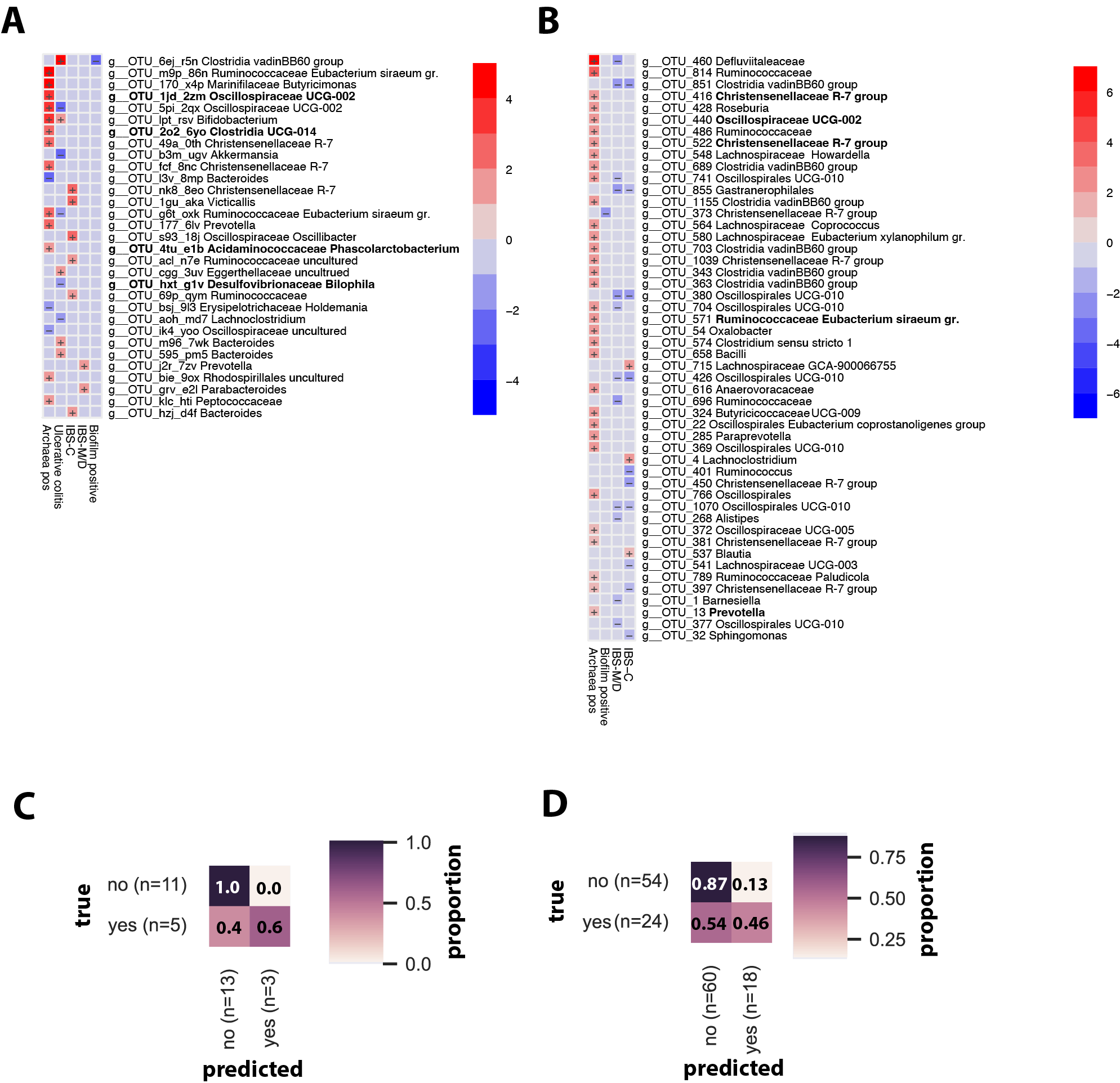


**Supplementary Figure 2 – Linear model and machine learning classification of microbiota data from cohort 1 and 2.** (A,B) Linear models generated with MaAsLin2: fecal microbiome composition ~ presence of arachae + disease + presence of endoscopic biofilms, Cohort 1 (A), Cohort 2 (B). (C-D) Machine learning classification of archaeal presence using qiime2 scikit-learn, based on fecal microbiome (archaea pos, yes) and negative (archaea neg, no). (C) Accuracy of the trained classifier with unseen samples from Cohort 1, overall accuracy = 88 %. (D) Accuracy of the trained classifier in Cohort 2, overall accuracy = 74 %. Statistical analysis: Cohort 1 total n=76 stool samples (50 archaea-neg, 26 archaea-pos; 23 controls, 34 IBS-M/D, 3 IBS-C and 16 UC-patients). Cohort 2 total n=78 stool samples (54 archaea-neg, 24 archaea-pos; 17 controls, 41 IBS-M/D, 18 IBS-C and 2 UC-patients).


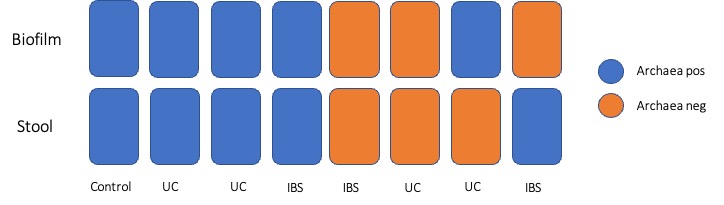


**Supplementary Figure 3. Matching stool and biofilms samples from the same individuals.** The match in archaea-status between stool and biofilm was 75%. Biofilm samples are depicted above, stool samples are below.

**Supplementary Methods**

**Fecal and biofilm DNA extraction with the use of Qiagen QIAamp DNA stool kit**

1. Homogenize the 150 to 200mg frozen feces with 1.0mL ASL lysis buffer of the kit by vortexing for 2 min in a 2mL tube containing 0.3g of sterile zirconia beads Ø 0,1mm zirconia (BioSpec, Cat. No. 11079101z). [if buffer shows precipitate, heat at 70°C before use]

2. Incubate for 15min at 95°C.

3. Cells are mechanically lysed by running the Fastprep™ Instrument for 1 min of bead beating at 6500 rpm and resting 5 min. Repeat 7 times.

4. Samples are allowed to cool down on ice for 2 min.

5. Samples are centrifuged at 16000 x g, 4°C, for 5 min.

6. Supernatant is transferred to a new 2mL tube.

7. The pellet is mixed with 300μL ASL lysis buffer of the kit, and steps 2-5 are repeated.

8. Supernatants are pooled in the new 2mL tube.

9. Add 260μl of 10M ammonium acetate to each lysate tube, mix well, and incubate on ice for 5 min.

10. Centrifuge at 16000 g, 4°C, for 10 min.

11. Transfer the supernatant to two 1.5mL Eppendorf tubes, add one volume of isopropanol, mix well, and incubate on ice overnight.

12. Centrifuge at 16000 g, 4°C, 15min, remove the supernatant using aspiration, wash nucleic acids pellet with 70 % EtOH (0,5mL) and dry the pellet under vacuum for 3 min.

13. Dissolve the nucleic acid pellet in 100μL of AE-Buffer and pool the two aliquots.

14. Add 2μL of DNase-free RNase (10mg/mL) and incubate at 37°C, 15 min.

15. Add 15μL proteinase K and 200μL AL buffer to the supernatant, vortex for 15 sec and incubate at 70°C for 10 min.

16. Add 200μL of ethanol (96-100%) to the lysate, and mix by vortexing.

17. Transfer to a QIAamp spin column and centrifuge at 16000 g for 1 min, at room temperature (RT).

18. Discard flow through, add 500μL buffer AW1 (Qiagen) and centrifuge at 16000 g for 1 min, at RT.

19. Discard flow through, add 500μL buffer AW2 (Qiagen) and centrifuge at 16000 g for 3 min, at RT

20. Dry the column by centrifugation at RT for 3.5 min.

21. Add 200μL Buffer AE (Qiagen), incubate for 2 min at RT

22. Centrifuge for 1 min at 16000 g to elute DNA.

Quality control: use 1% agarose gel

Sample concentration: use Nanodrop or Qubit

**Fungal PCR**

| **Primer name** | **Primer sequence** |
| --- | --- |
| ITS1-30F | GTCCCTGCCCTTTGTACACA |
| ITS1-217R | TTTCGCTGCGTTCTTCATCG |

| **Established PCR methods** | |
| --- | --- |
| **Reagents** | **Concentration** |
| DNA template | 100 ng |
| Primer ITS1-30F | 0.2 µM |
| Primer ITS1-217R | 0.2 µM |
| 2X GoTaq Green Master Mix | 12.5 µl |
| **Time** | **Temperature** |
| 3 min | 95° C |
| **40 Cycles of** | |
| 30 sec | 95° C |
| 30 sec | 55° C |
| 2 min | 72° C |
| 10 min | 72° C |
| ∞ | 12° C |

All PCR products were evaluated by 2 % agaroses-gel electrophoresis (Biozym LE Agarose, Biozym Scientific, Germany), using 1 µL of GelRed/ 100 µL (Biotium, USA) and ChemiDoc MP Imaging System (Bio-Rad, UK) to detect PCR product bands.

**Archaeal PCR**

In order to detect archaeal DNA, we performed nested PCR targeting the archaeal 16S rRNA gene. The first PCR was conducted using the primer 344aF and 1041R, which are archaea-specific and therefore increase archaeal DNA abundance compared to interfering bacterial DNA. In the second PCR, the primers Illu 519F and Illu 806R were used, which amplify universal 16S rRNA genes, also present in bacteria. Due to first increasing archaeal 16S amplicons, archaeal 16S is highly abundant and therefore it is unlikely that 16S of other origin is amplified.

For both PCRs, TAKARA Ex Taq buffer with MgCl2 (Takara Bio Inc., Tokyo, Japan), BSA (Roche Lifescience, Basel, Switzerland), dNTP mix, TAKARA Ex Taq Polymerase and water (Lichrosolv®; Merck, Darmstadt, Germany) were used.

| **Primer name** | **Primer sequence** |
| --- | --- |
| 344aF | ACGGGGYGCAGCAGGCGCGA |
| 1041R | GGCCATGCACCWCCTCTC |
| Illu 519F | CAGCMGCCGCGGTAA |
| Illu 806R | GGACTACVSGGGTATCTAAT |

| **Reagents** | **Concentration** |
| --- | --- |
| DNA template | 5 µl |
| Primer 344aF | 10 µM |
| Primer 1041R | 10 µM |
| TAKARA ExTaq Buffer with MgCl_2_ | 10 X |
| BSA | 20 mg/ml |
| dNTP mix | 2.5 mM |
| ExTaq Polymerase | 5 U/µl |
| **Time** | **Temperature** |
| 5 min | 95° C |
| **25 Cycles of** | |
| 30 sec | 94° C |
| 45 sec | 56° C |
| 1 min | 72° C |
| 10 min | 72° C |

| **Reagents** | **Concentration** |
| --- | --- |
| DNA template | 5 µl |
| Primer 519F | 10 µM |
| Primer 806R | 10 µM |
| TAKARA ExTaq Buffer with MgCl_2_ | 10 X |
| dNTP mix | 2.5 mM |
| Ex Taq Polymerase | 5 U/ µl |
| **Time** | **Temperature** |
| 5 min | 95° C |
| **40 Cycles of** | |
| 40 sec | 95° C |
| 2 min | 63° C |
| 1 min | 72° C |
| 10 min | 72°C |

PCR products were evaluated by 2 % agaroses-gel electrophoresis (Biozym LE Agarose, Biozym Scientific, Germany), using 1 µL of GelRed/ 100 µL (Biotium, USA) and ChemiDoc MP Imaging System (Bio-Rad, UK) to detect PCR product bands.
